# Serum uric acid to creatinine ratio in patients with early-onset post-stroke cognitive impairment: a retrospective cohort study

**DOI:** 10.3389/fnagi.2025.1580722

**Published:** 2025-07-02

**Authors:** Libin Liao, Weiquan Huang, Rongchao Ma, Wentong Hu, Hui Wu, Moxi Su, Dujuan Sha

**Affiliations:** ^1^Nanjing Drum Tower Hospital Clinical College of Nanjing Medical University, Nanjing, Jiangsu, China; ^2^Department of General Practice, Nanjing Drum Tower Hospital, Affiliated Hospital of Medical School, Nanjing University, Nanjing, China; ^3^State Key Laboratory of Pharmaceutical Biotechnology, Institute of Functional Biomolecules, Nanjing University, Nanjing, China

**Keywords:** serum uric acid to serum creatinine ratio, acute ischemic stroke, post-stroke cognitive impairment, biomarker, early-onset

## Abstract

**Background:**

Cognitive impairment is the major complication of acute ischemic stroke, which is a significant health concern imposing a heavy economic burden on families and society. Studies have shown that the serum uric acid (SUA) level is correlated to clinical outcomes of stroke and neurogenerative diseases. The serum uric acid to serum creatinine ratio (SUA/SCr) is an independent risk factor for poor outcomes of acute ischemic stroke and can potentially become an effective diagnostic indicator for cognitive decline. In this study, we aimed to investigate the association between SUA/SCr and early-onset post-stroke cognitive impairment.

**Methods:**

Consecutive acute ischemic stroke patients from our hospital were enrolled between June 2023 and September 2024. All blood samples were collected within 24 h after admission, and the cognitive function of patients was assessed within 2 weeks using the Chinese version of the Montreal Cognitive Assessment (MoCA). SUA/SCr was calculated by serum uric acid (umol/L)/serum creatinine (umol/L) and was split into three layers according to tertiles. The subjects were divided into a post-stroke cognitive impairment group and a non-post-stroke cognitive impairment group based on cognitive assessment. Binary logistic regression with different models, multivariate logistic regression analysis, and receiver operating characteristic (ROC) curves were adopted to evaluate the predictive ability of SUA/SCr in early-onset post-stroke cognitive impairment.

**Results:**

The current study showed that the post-stroke cognitive impairment group had lower SUA/SCr (*p* = 0.005) and the lower tertile of SUA/SCr is associated with a higher prevalence of post-stroke cognitive impairment (*p* = 0.008). The multivariate logistic analysis indicated that SUA/SCr (OR = 0.560, 95% CI = 0.321–0.976, *p* = 0.024) was independently associated with early-onset post-stroke cognitive impairment, and the lowest tertile was independently associated with a 5.903-fold increased risk of post-stroke cognitive impairment after adjusting for confounders. The optimal cutoff value of SUA/SCr to predict post-stroke cognitive impairment was 4.874, which gave a sensitivity of 72.22% and a specificity of 63.16%.

**Conclusion:**

Our study revealed that SUA/SCr can be a potential indicator for post-stroke cognitive impairment in the early phase, a lower level of SUA/SCr upon admission was independently correlated to cognitive dysfunction after stroke.

## 1 Introduction

Epidemiological studies have shown that stroke is one of the leading causes of death and disability globally, especially in Asia, where it is characterized by high prevalence, high morbidity, high mortality, and a heavy burden of disease (Tan et al., 2024). Stroke is the second leading cause of cognitive impairment with patients who have had a stroke often suffering severe mental deficits (Kaddumukasa et al., 2023). Post-stroke cognitive impairment (PSCI) is a common complication of stroke with a high prevalence and more than one-third of stroke survivors have PSCI (Chi et al., 2023). PSCI is defined as cognitive dysfunction following a stroke event at least 3–6 months, which covers a broad spectrum from mild cognitive decline to dementia (Huang et al., 2022) and is characterized by memory, executive function, learning, attention, decision-making, and spatial ability difficulties (Wang et al., 2024; Yu et al., 2024). Common vascular factors such as hypertension, atherosclerosis, diabetes mellitus, cerebrovascular diseases, smoking, and physical inactivity contribute to the progression of cognitive dysfunction after the patients suffer a stroke (Cuartero et al., 2023; Chang Wong and Chang Chui, 2022). A great number of factors such as neuroinflammation, oxidative stress (Lu et al., 2024), endothelial dysfunction, blood–brain barrier breakdown, and chronic cerebral hypoperfusion by some mechanistic and structural changes including energy imbalance, oxidative stress, endoplasmic reticulum stress, inflammation, and mitochondrial dysfunction (Rajeev et al., 2022), which are all involved in the pathophysiology of PSCI.

Early-onset cognitive impairment after acute ischemic stroke (AIS) usually leads to rapid changes in indicators. Some studies focus on the time within 2 weeks after stroke and define early-onset post-stroke cognitive impairment as the onset of cognitive impairment within 2 weeks of stroke (Xu et al., 2022; Xu et al., 2022). According to the previous survey, the Montreal Cognitive Assessment (MOCA) administered 7 days after stroke, predicts long-term cognitive function, functional outcome, and morbidity (Zietemann et al., 2018). Furthermore, MOCA as a brief tool is more sensitive to screening patients with mild cognitive impairment and better detects cognitive heterogeneity than other cognitive screening test tools (Jia et al., 2021).

Uric acid as a form of nitrogenous waste and the final product of purine metabolism, not only contributes to the formation of kidney stones but also has an association with the progression of chronic kidney diseases (Gherghina et al., 2022). Serum creatinine is a metabolite of human muscle closely related to estimated glomerular filtration rate (eGFR) (Gu et al., 2022). The serum uric acid to serum creatinine ratio (SUA/SCr) has been extensively studied in various diseases. It is commonly recognized as associated with decreased eGFR, renal function decline (Kawamoto et al., 2019), and an independent risk factor for diabetic kidney disease (Chen et al., 2022). Similarly, SUA/SCr is a good indicator for predicting non-alcoholic fatty liver disease (NAFLD) (Aktas et al., 2023), and incident cardiovascular disease (Wang et al., 2021).

Recent studies have shown that SUA/SCr is strongly associated with neurological disorders. Emerging studies support that a lower SUA/SCr ratio in patients with Parkinson’s disease (PD), also has a negative association with PD staging (Songsomboon et al., 2020). More importantly, it was proven to be positively correlated to the risk of stroke recurrence among young people with acute stroke attacks (Sun et al., 2022), and lower SUA/SCr with poor functional outcomes in acute ischemic stroke patients (Xu et al., 2024). Research has indicated that lower SUA/SCR ratios were associated with an increased risk of higher cognitive impairment among older adults aged 60 and above in the United States (Chen et al., 2024). Similarly, another piece of evidence from the China Health and Retirement Longitudinal Study showed that SUA/SCR holds promise as a potential indicator of cognitive decline in middle-aged adults (Zhou et al., 2025).

The predictors for cognitive decline after stroke are continuously researched, including the subject, inaccurate psychological testing, and inadequate diagnostic and prognostic value for PSCI. However, the relationship between SUA/SCr and patients with post-stroke cognitive impairment, especially in the early phase, has not been elucidated. Therefore, the objective of this study was to determine the potential association between the SUA/SCr ratio and people with cognitive impairment in the early stage of the acute ischemic attack.

## 2 Materials and methods

### 2.1 Study design and population

This cross-sectional retrospective cohort study was undertaken by patients with AIS admitted within 2 weeks to The Nanjing Drum Tower Hospital affiliated with Nanjing University Medical School between June 2023 and September 2024. All patients are scanned by computerized tomography (CT) or magnetic resonance imaging (MRI) to confirm the diagnosis of AIS.

**Figure 1:**
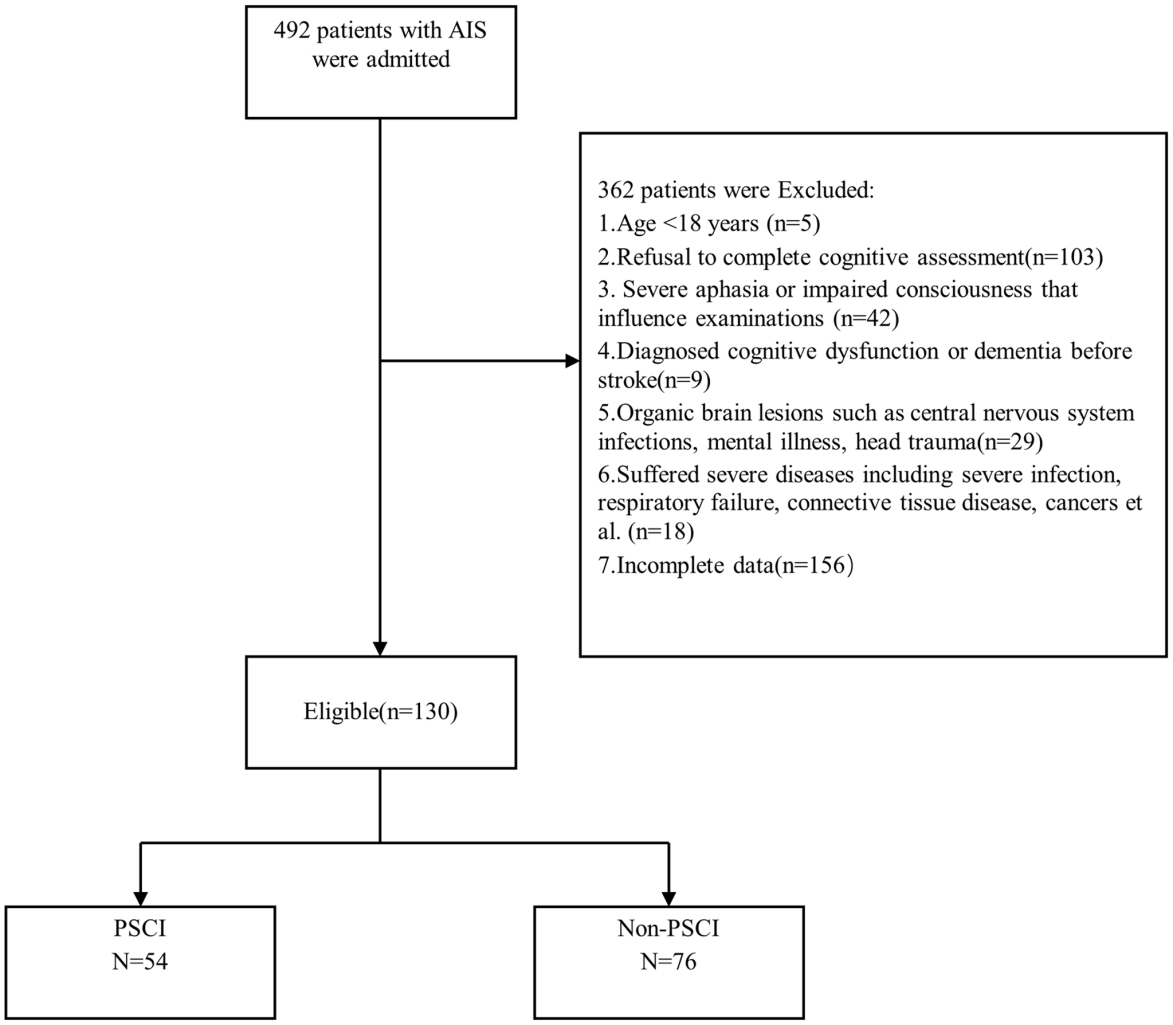
Flowchart of participant recruitment. PSCI, post-stroke cognitive impairment.

The inclusion criteria were adult patients with acute ischemic stroke. The exclusion criteria included the following: (1) <18 years of age, (2) the participants or their family refused cognitive assessment, (3) Severe aphasia or impaired consciousness that may influence examinations, (4) Participants diagnosed cognitive dysfunction or dementia before the stroke, (5) Organic brain lesions such as central nervous system infections, mental illness, head trauma, (6) Suffered severe diseases including severe infection, respiratory failure, connective tissue disease, cancer et al. (7) Incomplete data (shown in Figure 1).

All the procedures were carried out according to the ethical standards of the 1975 Declaration of Helsinki as proved by the Ethics Committee of The Nanjing Drum Tower Hospital (No. 2022-333-01). Given the retrospective study, the informed consent was waived.

### 2.2 Data collection and clinical measurements

The clinical data of subjects were collected from demographic information, medical histories, Clinical characteristics, and laboratory data. Demographic information included age, sex, body mass index (BMI), systolic blood pressure, diastolic blood pressure, and education years (defined as illiterate who have not been educated, less than or equal to 6 years of education, more than 6 years of education). Medical histories included a history of hypertension, diabetes, diabetes mellitus, hyperlipidemia, coronary artery disease, atrial fibrillation, previous stroke, smoking, and drinking. The Clinical characteristics included National Institutes of Health Stroke Scale (NIHSS) on admission, Fazekas score, carotid plaque, and carotid artery stenosis.

CT or MRI was performed on patients within 72 h after admission. All patients underwent carotid ultrasound or carotid artery CTA to assess plaque and stenosis. We also categorized the stroke etiology by Tial of Org 10172 in Acute Stroke Treatment (TOAST) criteria, evaluated the cerebral vascular damage by applying the Fazekas score, and the stroke severity assessed via the NIHSS.

All blood samples were collected within 24 h following hospital admission and were tested in the hospital laboratory department. The SUA/SCr ratio was calculated by the following formula: serum uric acid (μmmol/L)/serum creatinine (μmmol/L).

### 2.3 Evaluation of cognitive function

The cognitive function was assessed within 2 weeks by trained neurologists, using the Chinese version of the Montreal Cognitive Assessment (MoCA). The total MOCA score was 30 points. Cognitive impairment was defined by a MOCA score <26, we added 1 point to the MOCA score in patients with less than 12 years of education and a MOCA score of less than 26 (Zietemann et al., 2018; Xu et al., 2023).

### 2.4 Statistical analysis

For continuous variables, data following normal distribution were reported as the mean ± standard deviation (SD), followed by skewed distribution presented as median (25 and 75% interquartile). Categorical data were presented as numbers and percentages (%). Independent samples t-tests, Mann–Whitney *U* test, or chi-square (*χ*^2^) tests were used to compare the baseline characteristics of the PSCI and non-PSCI groups. All of the patients were divided into three groups according to SUA/SCr tertile. Differences in baseline characteristics among SUA/SCr tertiles were conducted using analysis of variance (ANOVA), the Kruskal–Wallis test, or the chi-square (*χ*^2^) test. Binary logistic regression analysis was performed to evaluate the association of SUA/SCr and PSCI. All variables with a significant relationship at *p* < 0.1 in univariate analysis entered multivariable analysis. Multivariate logistic regression analysis was then applied to evaluate the independent impact of SUA/SCr on the occurrence of PSCI, and the corresponding odds ratios (ORs) and 95% CIs were calculated. Multi-model logistic regression was developed to exclude confounding factors from interfering with the study results. Model 1: adjusted for age and sex. Model 2: further adjusted for stroke, education, and hyperlipidemia. Model 3: further adjusted for NHISS, MOCA, High sensitive C-Reactive protein (hs-CRP), Lymphocytes, Fibrinogen, D-Dimer, eGFR. The relationship between the SUA/SCr ratio and PSCI was analyzed by binary logistic regression after adjusting for the confounding factors, as indicated in the forest plot. In addition, the predictive value of the SUA/SCr ratio for PSCI was identified by a receiver operating characteristic (ROC) curve. Statistical significance was defined as a *p* < 0.05 (Two-tailed). All data were analyzed using the SPSS 27.0 statistical software. Graphical representations were created using GraphPad Prism.

## 3 Results

### 3.1 Demographic characteristics of PSCI and non-PSCI

**Figure 2:**
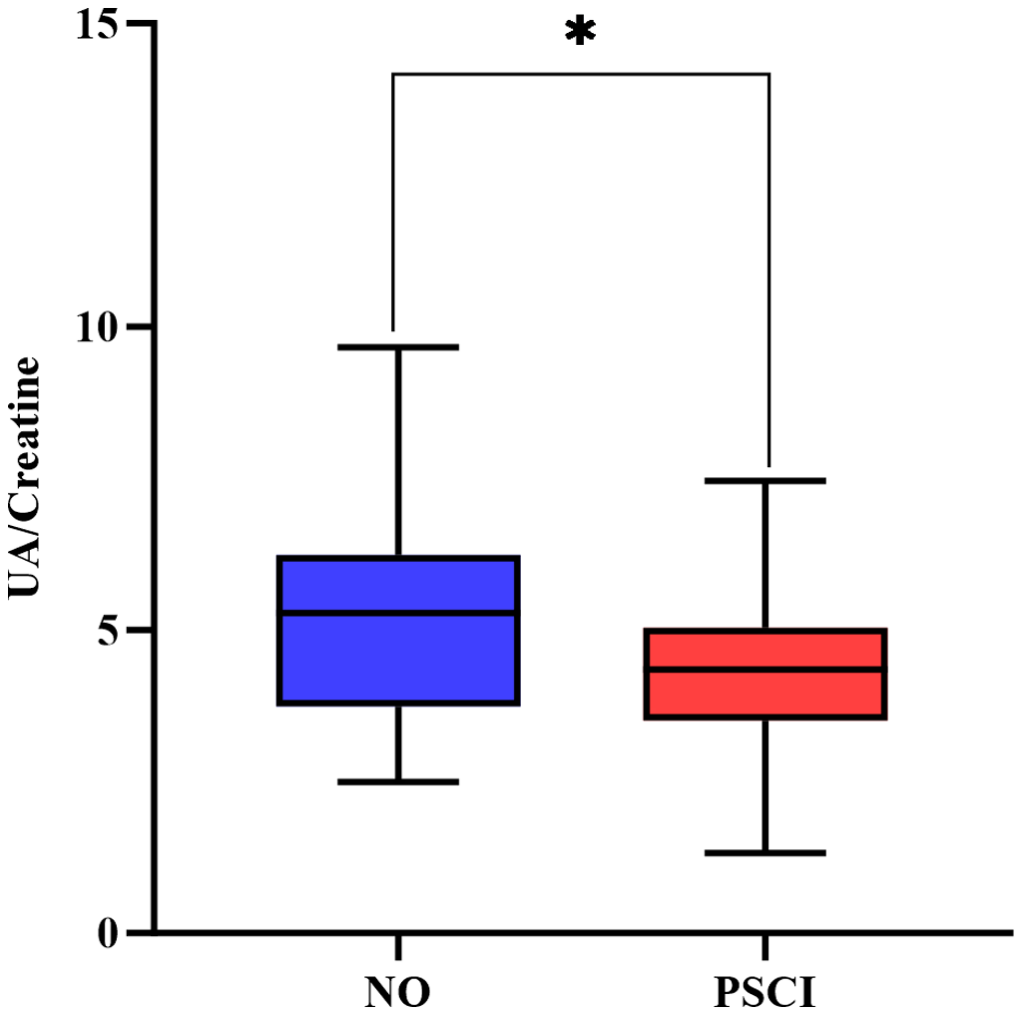
Box plots about the distribution of SUA/SCr in PSCI and non-PSCI subgroups. PSCI, post-stroke cognitive impairment. *P<0.05.

Among the 492 patients with acute ischemic stroke who were admitted, 130 patients were selected in the ultimate analysis. Of these individuals, 54 were in the PSCI group, and 76 in the non-PSCI group. The demographic data, medical history, clinical characteristics, and biochemical laboratory characteristics of PSCI and non-PSCI were summarized in Table 1. The results indicated that patients with PSCI have significantly higher levels in the incidence of stroke history, the NIHSS score on admission, fibrinogen (all *p* < 0.05), as well as lower levels in education level, MOCA scores, lymphocytes, triglyceride, total cholesterol, low-density cholesterol, C-Reactive Protein, and SUA/SCr ratio (all *p* < 0.05). Box plots about the distribution of SUA/SCr in subgroups are shown in Figure 2.

**Table 1:** Characteristics between the PSCI group and the non-PSCI group.

Variables	All patients (130)	PSCI (54)	Non-PSCI (76)	*p*-value
**Demographics**				
Male (*n* %)	89 (68.5)	37 (68.5)	52 (68.4)	0.991
Age (years), mean ± SD	63.85 (12.52)	65.22 (11.99)	62.87 (12.87)	0.293
BMI (kg/m^2^), mean ± SD	24.3 (2.94)	24.51 (2.89)	24.19 (2.96)	0.681
SBP (mmHg), mean ± SD	145.92 (21.31)	142.09 (22.21)	148.64 (20.36)	0.084
DBP (mmHg), mean ± SD	84.69 (13.04)	83.09 (14.07)	85.83 (12.24)	0.24
**Education**				0.012*
Illiterate, *n* (%)	7 (5.4)	3 (5.6)	4 (5.2)	
Years of education ≤ 6, *n* (%)	25 (19.2)	10 (18.5)	15 (19.7)	
Years of education > 6, *n* (%)	98 (75.4)	41 (75.9)	57 (75)	
**Medical history (*n* %)**				
Hypertension	86 (66.2)	40 (74.1)	46 (60.5)	0.108
Diabetes mellitus	38 (29.8)	15 (27.8)	23 (30.3)	0.759
Hyperlipidemia	43 (33.1)	18 (33.3)	25 (32.9)	0.958
Coronary artery disease	13 (10.2)	7 (13.5)	6 (8)	0.318
Atrial fibrillation	9 (7.1)	4 (7.7)	5 (6.7)	0.825
History of stroke	38 (29.2)	22 (40.7)	16 (21.1)	0.015*
Smoking	43 (33.1)	14 (25.9)	29 (38.2)	0.144
Drinking	21 (16.2)	8 (14.8)	13 (17.1)	0.727
**Clinical characteristics**				
NIHSS on admission (median, IQR)	3 (2–6)	4 (2–7)	2 (1–4)	0.002**
Fazekas score (median, IQR)	1 (1–2)	1 (1–2)	1 (1–2)	0.588
Carotid plaque, *n* (%)	95 (73.1)	41 (75.9)	54 (71.1)	0.537
Carotid artery stenosis, *n* (%)	52 (40)	27 (50)	25 (32.9)	0.05
**TOAST classification, *n* (%)**				0.66
Large atherosclerosis	57 (43.8)	22 (40.7)	35 (46.1)	
Small-vessel occlusion	25 (19.2)	9 (16.7)	16 (21.1)	
Cardioembolism	3 (2.3)	1 (1.9)	2 (2.6)	
Undetermined/unclassified	45 (34.6)	22 (40.7)	23 (30.3)	
**AIS hemisphere, *n* (%)**				0.898
Left	57 (43.8)	25 (46.3)	32 (42.1)	
Right	47 (36.2)	18 (33.3)	29 (38.2)	
Bilateral	20 (15.4)	9 (16.7)	11 (14.5)	
**MoCA (median, IQR)**	22.5 (19–26)	19 (12.75–21)	25 (23.25–27)	0.001**
**Laboratory characteristics**				
Leukocyte (10^9^/L) (median, IQR)	6.5 (5.4–8.25)	6.3 (5.4–8.3)	6.6 (5.35–8,2)	0.62
Neutrophils (10^9^/L) (median, IQR)	4.2 (3.28–5.45)	4 (3.1–5.63)	4.2 (3.4–5.1)	0.543
Lymphocytes (10^9^/L), mean (SD)	1.72 (0.54)	1.58 (0.46)	1.82 (0.57)	0.009**
Monocyte (10^9^/L) (median, IQR)	0.4 (0.3–0.5)	0.4 (0.3–0.6)	0.4 (0.3–0.5)	0.092
Platelets (10^9^/L) (median, IQR)	202 (159–239.25)	202 (151.75–243.25)	202 (168–238.5)	0.659
RDW (%) (median, IQR)	12.9 (12.5–13.38)	12.9 (12.5–13.4)	12.8 (12.5–13.4)	0.824
Fibrinogen (g/L) (median, IQR)	3 (2.5–3.6)	3.3 (2.9–3.88)	2.8 (2.4–3.4)	0.008**
D-dimer (mg/L) (median, IQR)	0.32 (0.21–0.8)	0.41 (0.25–0.96)	0.31 (0.19–0.71)	0.162
Albumin (g/L) (median, IQR)	39.45 (37.68–41.3)	39.2 (36.8–40.78)	39.85 (38.5–41.5)	0.159
FBG (mmol/L) (median, IQR)	5.23 (4.63–6.49)	5.47 (4.68–6.49)	5.21 (4.58–6.52)	0.55
HbA1c (%) (median, IQR)	5.9 (5.5–6.8)	5.9 (5.5–6.95)	5.9 (5.5–6.73)	0.929
Creatinine (umol/L) (median, IQR)	66.5 (52.75–78.25)	69 (52.75–82.25)	64.5 (54–77.75)	0.055
Uric acid (umol/L), mean (SD)	313.5 (104.38)	311.48 (96.82)	345.8 (107.8)	0.064
TG (mg/dL) (median, IQR)	1.32 (0.97–1.99)	1.19 (0.93–1.68)	1.50 (1.04–2.14)	0.039*
TC (mmol/L) (median, IQR)	4.52 (1.22)	4.18 (1.19)	4.76 (1.19)	0.007**
HDL-C (mmol/L) (median, IQR)	1.12 (0.89–1.32)	1.08 (0.85–1.3)	1.17 (0.92–1.39)	0.11
LDL-C (mmol/L) (mean ± SD)	2.71 (0.97)	2.44 (0.95)	2.91 (0.93)	0.005**
eGFR (mL/min/1.73 m^2^) (mean ± SD)	105.74 (31.37)	103.40 (34.53)	107.43 (29)	0.473
Hs-CRP (mg/L) (median, IQR)	4.1 (2.8–7.98)	3.7 (2.75–6.25)	5 (3–16.2)	0.022*
SUA/SCr (median, IQR)	4.85 (3.77–5.73)	4.40 (3.76–5.23)	5.27 (3.74–6.23)	0.005**

*p < 0.05, **p < 0.01, ***p < 0.001.

PSCI, post-stroke cognitive impairment; BMI, body mass index; SBP, systolic blood pressure; DBP, diastolic blood pressure; NIHSS, National Institutes of Health Stroke Scale; TOAST, Trial of Org 10172 in Acute Stroke Treatment; AIS, acute ischemic stroke; MOCA, Montreal Cognitive Assessment; RDW, red cell distribution width; FBG, fasting blood glucose; HbA1c, glycosylated hemoglobin A1c; TG, triglyceride; TC, total cholesterol; HDL-C, high-density lipoprotein cholesterol; LDL-C, low-density lipoprotein cholesterol; eGFR, estimated glomerular filtration rate; Hs-CRP, High sensitive C-Reactive protein; SUA/SCr, serum uric acid to serum creatinine ratio.

### 3.2 Demographic characteristics of all patients in SUA/SCr tertiles

**Figure 3:**
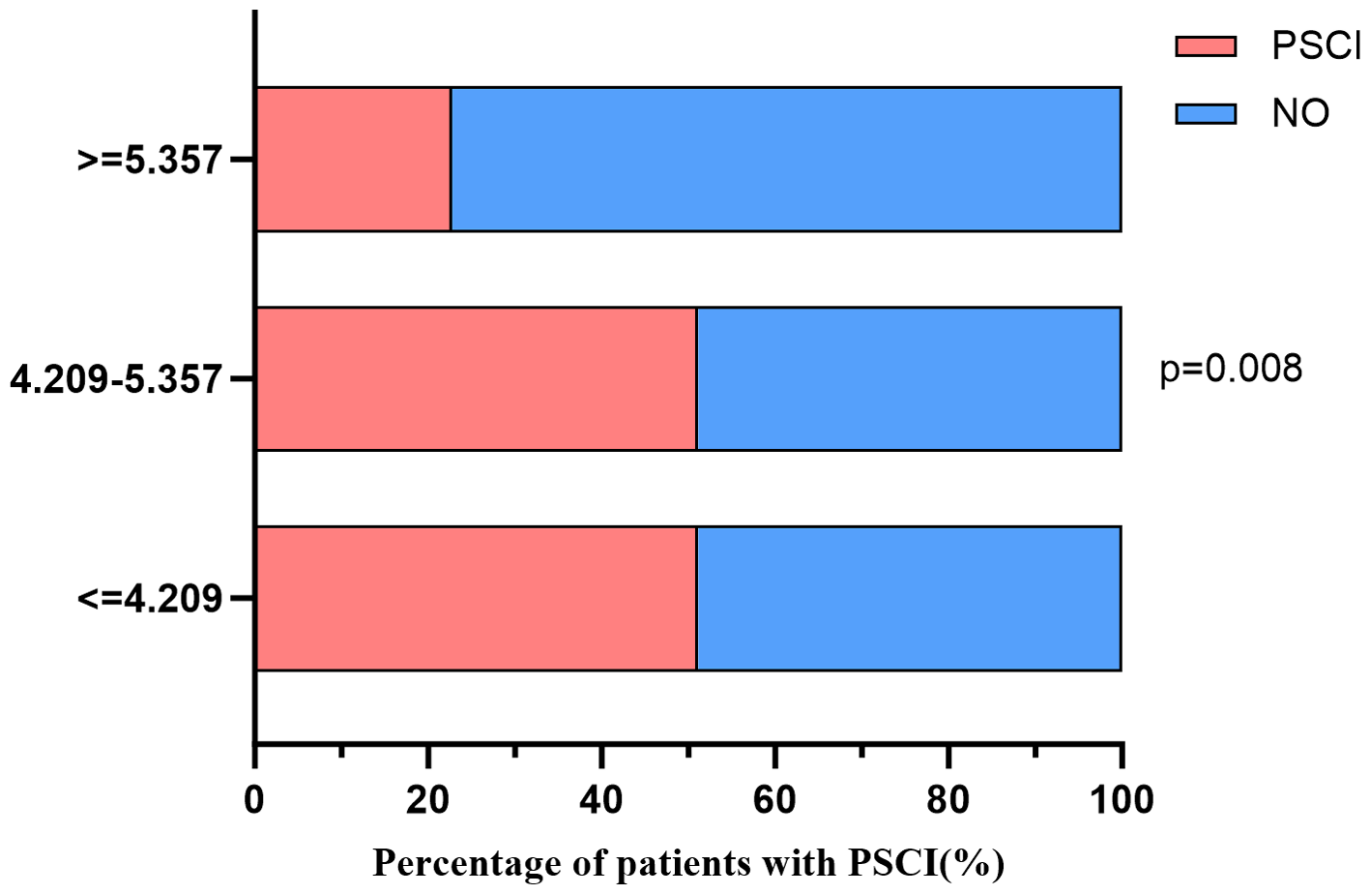
Percentage of PSCI occurrence according to SUA/SCr tertiles. PSCI, post-stroke cognitive impairment.

**Figure 4:**
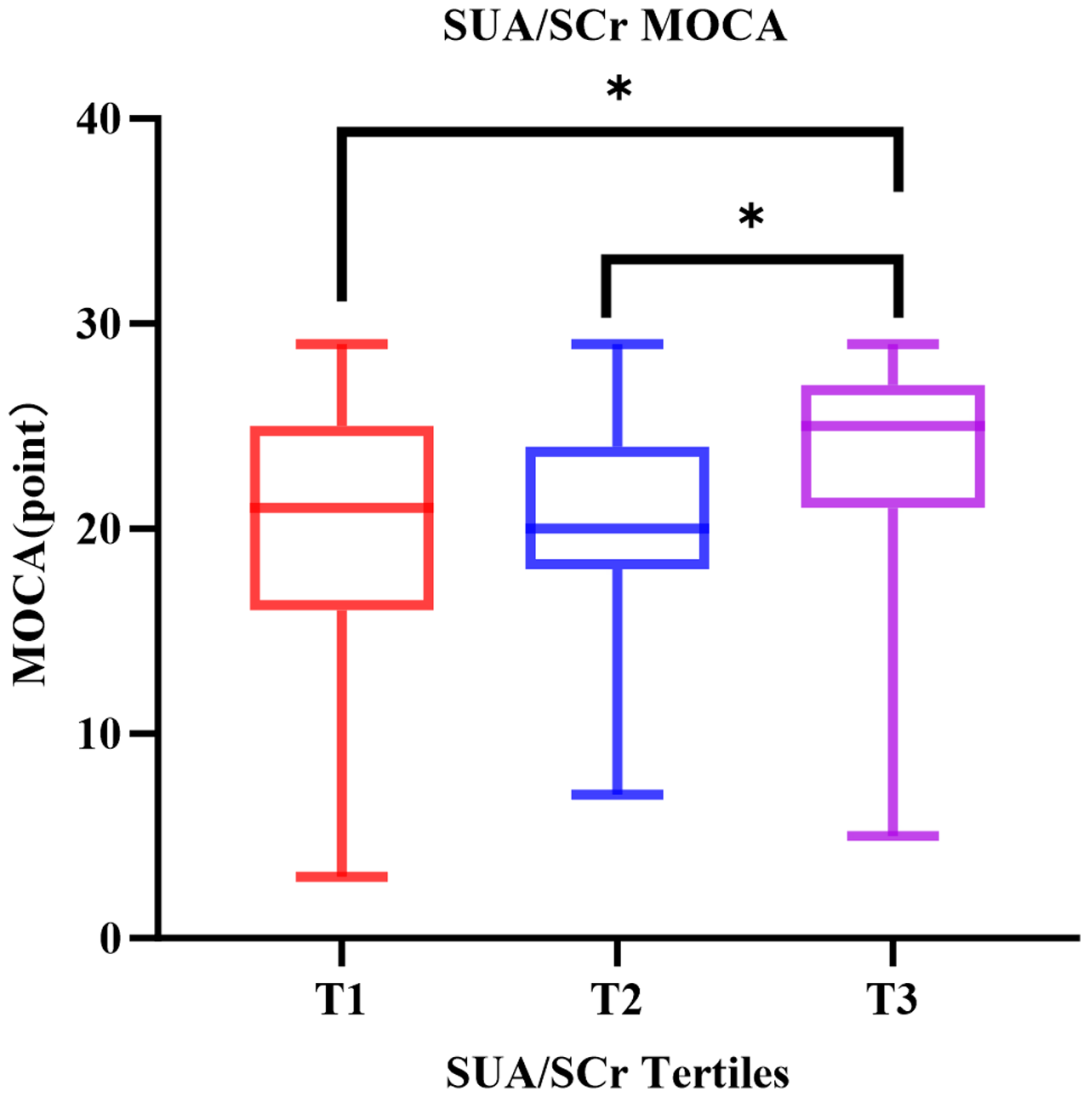
The distribution of MOCA scores among SUA/SCr tertiles. MOCA, Montreal Cognitive Assessment; SUA/SCr, serum uric acid to serum creatinine ratio. *P<0.05.

The 130 individuals were divided by SUA/Scr tertiles, 44 in group T1 (≤4.209), 42 in group T2 (4.210–5.356), and 44 in group T3 (≥5.357). The range of SUA/SCr in all patients was 1.367–9.657. Significant differences in proportions of PSCI and non-PSCI patients were observed in SUA/SCr tertiles, shown in Figure 3 (Tertile 1: 51.16%, Tertile 2: 51.16%, Tertile 3: 22.73%, *p* = 0.008). Meanwhile, we analyzed the MOCA scores in SUA/Scr tertiles and found that MOCA scores in the lowest and second tertiles were significantly different from the highest tertile (shown in Figure 4). The comparison of baseline characteristics among the three groups showed that age, hyperlipidemia incidence, NIHSS score on admission, MOCA score, lymphocytes, eGFR, uric acid, and creatinine have significant differences (all *p* < 0.05). In contrast, other variables did not differ (Table 2). Furthermore, those patients with a high SUA/SCr ratio were significantly associated with higher levels of eGFR and uric acid and lower levels of creatinine (all *p* < 0.001).

**Table 2:** Baseline characteristics of groups according to SUA/SCr tertiles.

Variables	SUA/SCr tertiles			*p*-value
	≤4.209 (44)	4.210–5.356 (42)	≥5.357 (44)	
**Demographics**				
Male (*n* %)	32 (74.4)	30 (69.8)	27 (61.4)	0.413
Age (years), mean ± SD	65.53 (10.08)	68.74 (11.5)	57.41 (13.14)	<0.001***
BMI (kg/m^2^), mean ± SD	23.37 (3.23)	24.28 (2.13)	25.26 (3.26)	0.125
SBP (mmHg), mean ± SD	142.53 (22.28)	148.42 (17.24)	146.8 (23.85)	0.42
DBP (mmHg), mean ± SD	82.51 (12.89)	85.47 (12.43)	86.07 (13.79)	0.401
**Education**				0.219
Illiterate, *n* (%)	0	3 (7)	4 (9.1)	
Years of education ≤ 6, *n* (%)	7 (16.3)	11 (25.6)	7 (15.9)	
Years of education > 6, *n* (%)	36 (83.7)	29 (67.4)	33 (75)	
**Medical history (*n* %)**				
Hypertension	32 (74.4)	28 (65.1)	26 (59.1)	0.351
Diabetes mellitus	11 (25.6)	16 (37.2)	11 (25)	0.371
Hyperlipidemia	7 (16.3)	16 (37.2)	20 (45.5)	0.012*
Coronary artery disease	4 (9.3)	7 (16.3)	2 (4.5)	0.186
Atrial fibrillation	3 (7)	5 (11.6)	1 (2.3)	0.228
History of stroke	13 (30.2)	12 (27.9)	13 (29.1)	0.971
Smoking	12 (27.9)	13 (30.2)	18 (40.9)	0.388
Drinking	5 (11.6)	6 (14)	10 (22.7)	0.332
**Clinical characteristics**				
NIHSS on admission (median, IQR)	4 (2–7)	3 (2–5)	2 (1–4)	0.049*
Fazekas score (median, IQR)	1 (1–2)	1 (1–2)	1 (1–1)	0.157
Carotid plaque, *n* (%)	34 (79.1)	33 (76.7)	28 (63.6)	0.215
Carotid artery stenosis, *n* (%)	11 (25.6)	22 (51.2)	19 (43.2)	0.046*
**TOAST classification, *n* (%)**				0.085
Large atherosclerosis	12 (27.9)	20 (46.5)	25 (58.9)	
Small-vessel occlusion	8 (18.6)	10 (23.3)	7 (15.9)	
Cardioembolism	1 (2.3)	1 (2.3)	1 (2.3)	
Undetermined/unclassified	22 (51.2)	12 (27.9)	11 (25)	
**AIS hemisphere, *n* (%)**				0.953
Left	18 (41.9)	20 (46.5)	19 (43.2)	
Right	17 (39.5)	14 (32.6)	16 (36.4)	
Bilateral	6 (14)	8 (18.6)	6 (13.6)	
**MoCA (median, IQR)**	22 (16–25)	20 (17.75–24.25)	25 (21–27)	0.003**
**Laboratory characteristics**				
Leukocyte (10^9^/L) (median, IQR)	7.2 (5.5–9.2)	5.75 (5.175–7.65)	6.6 (5.675–7.725)	0.136
Neutrophils (10^9^/L) (median, IQR)	4.8 (3.1–6.2)	4.0 (3.1–5.125)	4.15 (3.575–4.9)	0.123
Lymphocytes (10^9^/L), mean (SD)	1.674 (0.54)	1.591 (0.519)	1.183 (0.514)	0.024*
Monocyte (10^9^/L) (median, IQR)	0.4 (0.3–0.6)	0.4 (0.275–0.5)	0.4 (0.3–0.4)	0.116
Platelets (10^9^/L) (median, IQR)	194.5 (156.75–224.25)	197.5 (156.5–249)	214 (173–240)	0.381
RDW (%) (median, IQR)	12.7 (12.5–13.1)	13.0 (12.475–13.625)	12.85 (12.5–13.125)	0.316
Fibrinogen (g/L) (median, IQR)	3.2 (2.4–3.8)	3.15 (2.7–3.6)	2.9 (2.475–3.45)	0.574
D-dimer (mg/L) (median, IQR)	0.43 (0.26–0.92)	0.415 (0.228–0.843)	0.285 (0.15–0.695)	0.063
Albumin (g/L) (median, IQR)	39 (37.3–41)	39.3 (37.55–40.625)	40.65 (38.525–42.075)	0.159
FBG (mmol/L) (median, IQR)	5.12 (4.54–6.48)	5.525 (4.623–7.24)	5.335 (4.855–6.295)	0.61
HbA1c (%) (median, IQR)	5.95 (5.57–7.58)	6 (5.6–7.03)	5.8 (5.5–6.5)	0.369
TG (mg/dL) (median, IQR)	1.19 (0.96–1.59)	1.18 (0.95–2.05)	1.15 (1.135–2.463)	0.078
TC (mmol/L) (median, IQR)	4.02 (3.48–4.83)	4.62 (3.95–5.53)	4.655 (3.598–5.528)	0.081
HDL-C (mmol/L) (median, IQR)	1.08 (0.89–1.30)	1.14 (0.953–1.403)	1.07 (0.87–1.30)	0.593
LDL-C (mmol/L), mean (SD)	2.48 (0.89)	2.76 (1.00)	2.90 (0.97)	0.126
eGFR (mL/min/1.73 m^2^), mean (SD)	90.71 (28.64)	104.85 (32.90)	121.29 (24.92)	<0.001***
Hs-CRP (mg/L) (median, IQR)	4.6 (3.8–16.2)	3.8 (2.7–7.7)	3.95 (2.725–5.45)	0.349
Uric acid (umol/L), mean (SD)	265.67 (79.51)	332.6 (86.88)	394.89 (103.41)	<0.001***
Creatinine (umol/L) (median, IQR)	75 (62–89)	66.5 (55.75–78.5)	55 (49–70)	<0.001***

*p < 0.05, **p < 0.01, ***p < 0.001.

SUA/SCr, serum uric acid to serum creatinine ratio; BMI, body mass index; SBP, systolic blood pressure; DBP, diastolic blood pressure; NIHSS, National Institutes of Health Stroke Scale; TOAST, Trial of Org 10172 in Acute Stroke Treatment; AIS, acute ischemic stroke; MOCA, Montreal Cognitive Assessment; RDW, red cell distribution width; FBG, fasting blood glucose; HbA1c, glycosylated hemoglobin A1c; TG, triglyceride; TC, total cholesterol; HDL-C, high-density lipoprotein cholesterol; LDL-C, low-density lipoprotein cholesterol; eGFR, estimated glomerular filtration rate; Hs-CRP, High sensitive C-Reactive protein.

### 3.3 Relationship between SUA/SCr and early-onset PSCI

When taking SUA/SCr as a continuous variable, the logistic regression analysis results for the risk factors associated with early-onset PSCI are shown in Table 3. The univariate logistic regression analysis presented that stroke history, NIHSS score on admission, MOCA score, lymphocytes, fibrinogen, D-dimer, total cholesterol, low-density-lipoprotein, eGFR, hs-CRP, and SUA/SCr are all associated with early-onset PSCI (all *p* < 0.05). All variables *p* < 0.1 entered into multivariate logistic regression analysis, which showed that MOCA (OR = 0.646, 95%CI = 0.528–0.781, *p* < 0.001) and SUA/SCr (OR = 0.560, 95%CI = 0.321–0.976, *p* = 0.024) were independently associated with early-onset PSCI. To further investigate the relationship between SUA/SCR and PSCI, we developed 3 models, including statistically significant covariates and clinically significant models (Table 4). SUA/SCr ratio was entered as a categorical variable into the model for analysis, and *p*-values were obtained for the comparison. In comparison to Tertile 3 (≥5.357), the lowest tertile (≤4.209) was independently associated with a nearly 5.903-fold increased risk of PSCI after adjusted for potential confounders such as age, sex, stroke and hyperlipidemia history, education level, NIHSS score, MOCA score, hs-CRP, Lymphocytes, Fibrinogen, D-Dimer, and glomerular filtration rate.

**Table 3:** Univariate and multivariate analyses for the potential predictive factors associated with PSCI with logistic regression analysis.

Variables	Univariate analysis		Multivariate analysis	
	OR (95%CI)	*p*-value	OR (95%CI)	*p*-value
**Demographics**				
Male (*n* %)	1.005 (0.474–2.128)	0.991		
Age (years), mean ± SD	1.015 (0.987–1.045)	0.291		
BMI (kg/m^2^), mean ± SD	1.039 (0.869–1.241)	0.676		
SBP (mmHg), mean ± SD	0.985 (0.969–1.002)	0.087	0.973 (0.941–1.007)	0.118
DBP (mmHg), mean ± SD	0.984 (0.957–1.011)	0.239		
**Education**				
Illiterate, *n* (%)	Reference			
Years of education ≤ 6, *n* (%)	0.889 (0.163–4.853)	0.892		
Years of education > 6, *n* (%)	0.959 (0.204–4.518)	0.958		
**Medical history (*n* %)**				
Hypertension	1.863 (0.869–3.996)	0.11	1.782 (0.420–7.563)	0.434
Diabetes mellitus	0.886 (0.410–1.916)	0.759		
Hyperlipidemia	1.020 (0.486–2.140)	0.958		
Coronary artery disease	1.738 (0.549–5.495)	0.347		
Atrial fibrillation	1.136 (0.290–4.442)	0.855		
History of stroke	2.578 (1.189–5.589)	0.016*	1.636 (0.455–5.879)	0.451
Smoking	0.567 (0.264–1.219)	1.219		
Drinking	0.843 (0.323–2.200)	0.727		
**Clinical characteristics**				
NIHSS on admission	1.181 (1.046–1.334)	0.007**	0.844 (0.644–1.074)	0.168
Fazekas score	1.065 (0.644–1.760)	0.807		
Carotid plaque, *n* (%)	1.285 (0.579–2.851)	0.537		
Carotid artery stenosis, *n* (%)	2.040 (0.996–4.177)	0.051	1.744 (0.448–7.024)	0.414
**TOAST classification, *n* (%)**				
Large atherosclerosis	Reference			
Small-vessel occlusion	1.257 (0.108–14.700)	0.855		
Cardioembolism	1.125 (0.089–14.202)	0.927		
Undetermined/unclassified	1.913 (0.162–22.630)	0.607		
**AIS hemisphere, *n* (%)**				
Left	Reference			
Right	0.955 (0.343–2.660)	0.93		
Bilateral	0.759 (0.263–2.188)	0.609		
MoCA (median, IQR)*	0.640 (0.547–0.749)	<0.001***	0.646 (0.528–0.781)	<0.001***
**Laboratory characteristics**				
Leukocyte (10^9^/L) (median, IQR)	0.973 (0.845–1.121)	0.708		
Neutrophils (10^9^/L) (median, IQR)	0.987 (0.844–1.155)	0.874		
Lymphocytes (10^9^/L), mean (SD)	0.392 (0.190–0.809)	0.011*	0.547 (0.141–2.114)	0.382
Monocyte (10^9^/L) (median, IQR)	8.139 (0.875–75.709)	0.065	0 (0–25.204)	0.127
Platelets (10^9^/L) (median, IQR)	1.000 (0.994–1.006)	0.978		
RDW (%) (median, IQR)	1.249 (0.868–1.796)	0.231		
Fibrinogen (g/L) (median, IQR)	1.508 (1.049–2.166)	0.026*	0.850 (0.387–1.868)	0.686
D-dimer (mg/L) (median, IQR)	1.496 (1.006–2.225)	0.047	1.077 (0.476–2.439)	0.859
Albumin (g/L) (median, IQR)	0.927 (0.838–1.027)	0.147	1.027 (0.796–1.326)	0.08
FBG (mmol/L) (median, IQR)	1.030 (0.862–1.230)	0.743		
HbA1c (%) (median, IQR)	1.027 (0.831–1.269)	0.805		
Creatinine	1.007 (0.992–1.021)	0.362		
Uric acid (umol/L), mean (SD)	0.997 (0.993–1.000)	0.068	1.002 (0.995–1.009)	0.657
TG (mg/dL) (median, IQR)	0.741 (0.505–1.088)	0.126	2.269 (0.588–8.753)	0.234
TC (mmol/L) (median, IQR)	0.655 (0.477–0.898)	0.009**	0.361 (0.053–2.476)	0.3
HDL-C (mmol/L) (median, IQR)	0.429 (0.134–1.370)	0.153		
LDL-C (mmol/L), mean (SD)	0.576 (0.386–0.860)	0.007**	17.512 (0.712–430.971)	0.08
eGFR (mL/min/1.73 m^2^), mean (SD)	0.996 (0.985–1.007)	0.47		
Hs-CRP (mg/L) (median, IQR)	1.047 (1.010–1.085)	0.012*	1.080 (0.990–1.178)	0.083
SUA/SCr	0.718 (0.560–0.920)	0.009**	0.560 (0.321–0.976)	0.024*

*p < 0.05, **p < 0.01, ***p < 0.001.

BMI, body mass index; SBP, systolic blood pressure; DBP, diastolic blood pressure; NIHSS, National Institutes of Health Stroke Scale; TOAST, Trial of Org 10172 in Acute Stroke Treatment; AIS, acute ischemic stroke; MOCA, Montreal Cognitive Assessment; RDW, red cell distribution width; FBG, fasting blood glucose; HbA1c, glycosylated hemoglobin A1c; TG, triglyceride; TC, total cholesterol; HDL-C, high-density lipoprotein cholesterol; LDL-C, low-density lipoprotein cholesterol; eGFR, estimated glomerular filtration rate; Hs-CRP, High sensitive C-Reactive protein; SUA/SCr, serum uric acid to serum creatinine ratio.

**Table 4:** Risks for the incidence of PSCI development according to the tertiles of baseline SUA/SCr Levels.

Model	Tertile	OR	95%CI	*p*-value
Model 1	Tertile 1 (≤4.209)	3.689	1.398–9.733	0.008**
	Tertile 2 (4.209–5.357)	3.722	1.373–10.09	0.01*
	Tertile 3 (≥5.357)	Reference		
Model 2	Tertile 1 (≤4.209)	5.555	1.895–16.282	0.002**
	Tertile 2 (4.209–5.357)	4.779	1.65–13.84	0.004**
	Tertile 3 (≥5.357)	Reference		
Model 3	Tertile 1 (≤4.209)	5.903	1.103–34.417	0.048*
	Tertile 2 (4.209–5.357)	14.266	2.47–82.388	0.003**
	Tertile 3 (≥5.357)	Reference		

*p < 0.05, **p < 0.01, ***p < 0.001.

Reference (1.0) is the highest tertile of SUA/SCr tertile for PSCI.

Model 1: adjusted for age and sex.

Model 2: further adjusted for stroke, education, and hyperlipidemia.

Model 3: further adjusted for NHISS, MOCA, hs-CRP, Lymphocytes, Fibrinogen, D-Dimer, eGFR.

OR, odds ratio; CI, confidence interval; NIHSS, National Institutes of Health Stroke Scale; MOCA, Montreal Cognitive Assessment; SUA/SCr, serum uric acid to serum creatinine ratio; Hs-CRP, High sensitive C-Reactive protein; eGFR, estimated glomerular filtration rate.

**Figure 5:**
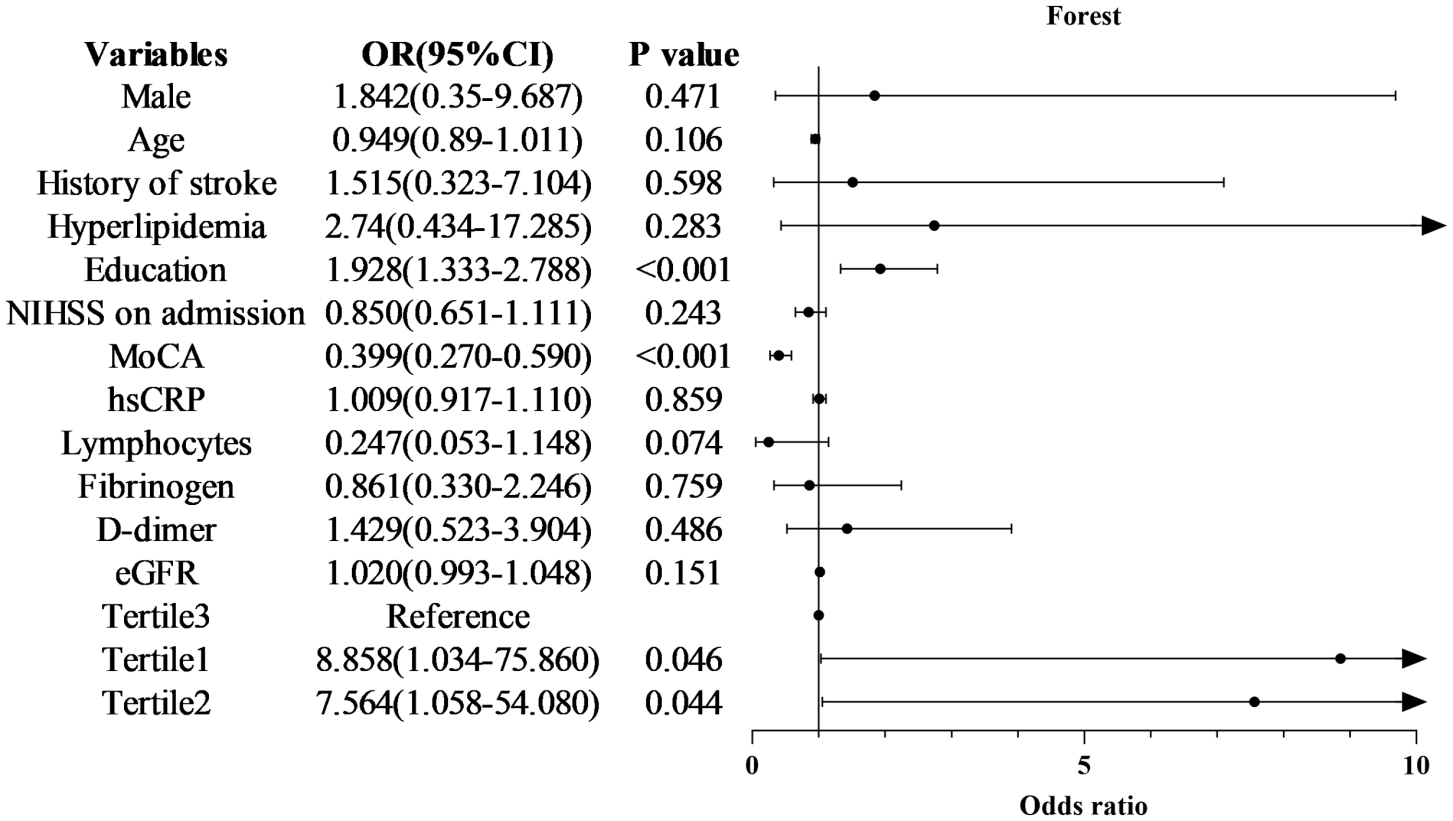
Forest plot of odds ratios for PSCI. NIHSS, National Institutes of Health Stroke Scale; MOCA, Montreal Cognitive Assessment; hs-CRP, High sensitive C-Reactive protein; eGFR, estimated glomerular filtration rate; PSCI, post-stroke cognitive impairment, CI, confidence interval.

The lowest tertile of the SUA/SCr ratio (≤4.209) was substantially related to a greater risk of PSCI (OR = 8.858, 95%CI = 1.034–75.86, *p* = 0.046), the middle tertile of the SUA/SCr (OR = 7.564, 95%CI = 1.058–54.080, *p* = 0.044), MOCA (OR = 0.399, 95%CI = 0.270–0.590, *p* < 0.001), and education (OR = 1.928, 95%CI = 1.333–2.788, *p* < 0.001) also showed significant association with the prevalence of early-onset PSCI (all *p* < 0.05), as indicated in the forest plot in Figure 5. The highest tertile served as a reference after adjusting the confounders such as age, sex, stroke and hyperlipidemia history, education level, NIHSS score, MOCA score, hs-CRP, Lymphocytes, Fibrinogen, D-dimer, and estimated glomerular filtration rate.

### 3.4 The predictive accuracy of the SUA/SCr ratio as a biomarker for PSCI

**Figure 6:**
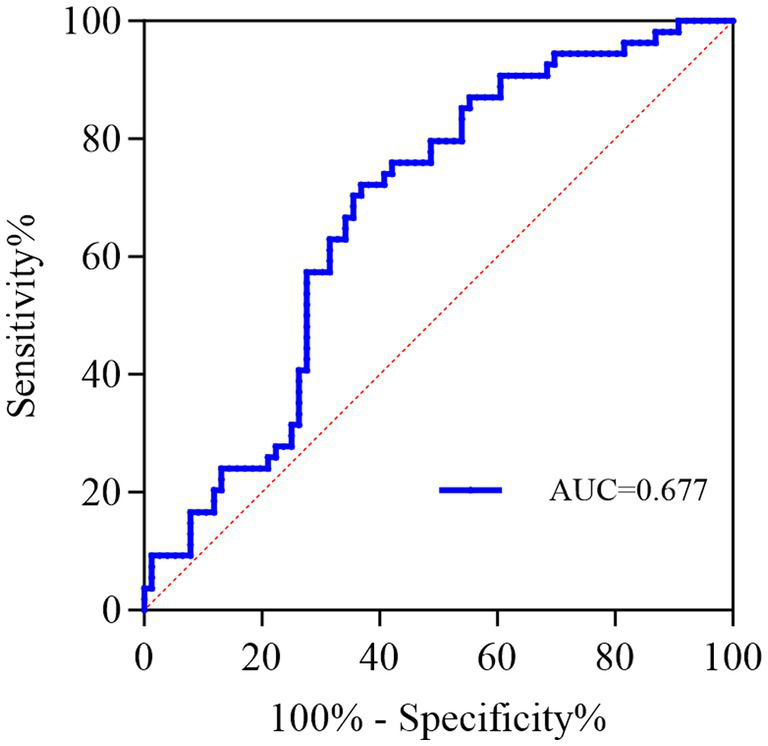
Receiver operating characteristic (ROC) curve for SUA/SCr as a predictor of PSCI. The corresponding area under the curve (AUC) to indicate PSCI was 0.677 (95%CI = 0.585–0.769, *p* = 0.0006). The optimal cutoff value of SUA/SCr for diagnosing PSCI was 4.874 with a sensitivity of 72.22% and a specificity of 63.16%.

ROC analysis was further conducted to explore the ability of the SUA/SCr ratio to predict the development of PSCI. The results showed that the corresponding area under the curve (AUC) to indicate PSCI was 0.677 (95%CI = 0.585–0.769, *p* = 0.0006). The optimal cutoff value of SUA/SCr for the diagnosis of PSCI was 4.874 with a sensitivity of 72.22% and a specificity of 63.16% (Figure 6).

## 4 Discussion

To the best of our knowledge, this was the first study to investigate the potential link between SUA/SCr and cognitive impairment following an acute ischemic stroke in the early phase of 2 weeks. In this study, we found a higher prevalence of PSCI associated with a decrease in the SUA/SCr ratio.

It is well known that uric acid is the final product of purine metabolites, and creatinine is a substance derived from muscle and a great indicator for evaluating renal function. Previous studies have reported that SUA is associated with the prognosis of stroke, although conclusions on prognosis are not consistent. Li et al. showed that high serum uric acid levels enhance the recurrence rate of stroke (Li et al., 2023). Similar results were observed in a prospective cohort study in which the risk of incident stroke was elevated with increasing cumulative uric acid (Tian et al., 2023). By contrast, Zhang et al. reported that for individuals with higher UA levels with better outcomes after AIS, a linear dose–response relationship exists between uric acid levels and the clinical outcome of patients with AIS (Zhang et al., 2024). There were many studies on the relationship between creatinine and stroke, but creatinine was often presented as a ratio (Satoh, 2022; Li et al., 2023). In addition, one Japanese observational study revealed that low creatinine clearance upon admission is a risk factor for unfavorable functional outcomes in female patients with cardioembolic stroke (Sakai et al., 2023). Although not statistically significant, our study found lower uric acid levels and higher creatinine levels in the PSCI group. Furthermore, SUA and creatinine both correlate with renal function. Several studies provided evidence that higher baseline SUA levels are correlated to an increased risk of chronic kidney disease and show a non-linear relationship (Luo et al., 2023). Baral et al. conducted an observational cross-sectional study on patients with diabetes, and the results showed that SUA/SCr served as a biomarker for renal injury had a positive correlation with glomerular filtration rate (Baral et al., 2023). Similar to their study, a positive and statistically significant correlation of eGFR with increasing UA tertiles was similarly found in our results.

The international Vascular Impairment of Cognition Classification Consensus Study (VICCCS) built the consensus for a vascular cognitive decline into mild and major, four major vascular impairment (VCI) sub-types including post-stroke dementia, subcortical ischemic vascular dementia, multi-infarct (cortical) dementia, and mixed dementias are defined (Rundek et al., 2022). PSCI presents up to nearly 50% in some countries and it is a common cause of long-term disability and reduced quality of life (Jimenez-Ruiz et al., 2024). Previous studies revealed that circulating biomarkers have significant value for predicting the occurrence and development of PSCI (Johansen et al., 2024; Tao et al., 2022). Yan et al. performed a meta-analysis to evaluate the association between SUA and PSCI, and the results showed that AIS patients with higher SUA had an increased risk of PSCI (Yan et al., 2022). Whether the high SUA/SCr level is favorable for stroke remains controversial. Recent studies have reported that higher SUA/SCr was associated with elevated risk of stroke, including ischemic stroke and hemorrhagic stroke (Wang et al., 2021). However, a multicenter study discovered that SUA/SCr had negative and non-linear associations with 1-year stroke recurrence, the study suggested that high SUA/SCr may be protective to stroke patients (Zhang et al., 2024). Guo et al. found a lower SUA/SCr ratio was associated with poor functional outcomes in patients who suffered from acute ischemic stroke at 3 months and 1 year (Gong et al., 2022). The latter two results are consistent with our study that high SUA/SCr may be a protective factor after stroke, especially in cognitive function. Chen et al. first investigated the negative and non-linear relationship between SUA/SCr and the risk of cognitive impairment among older American adults. They suggested that lower SUA/SCr had a higher risk of cognitive decline (Chen et al., 2024). Similarly, our study found that MOCA scores are statistically significant with a non-linear relationship in 3 tertiles of SUA/SCr and SUA/SCr is a promising indicator in patients with cognitive impairment after stroke.

The mechanisms that lead to increased risk of PSCI in lower SUA/SCr individuals are not completely understood. Some studies suggested that high uric acid levels mediated the development of cognitive impairment by activating the Toll-like receptor 4 (TLR4)/NF-ΚB signaling pathway, inflammation, oxidative stress, and endothelial dysfunction (Chen et al., 2024). What needs to be emphasized is that uric acid has both neuroprotective and damaging effects, and uric acid therapy may be a novel agent for cognitive dysfunction diseases (Zhu et al., 2024). Xiao et al. reported that UA upregulates the autophagy-related proteins such as Beclin-1, and LAMP-1 protects brain cells from toxicity, and activates microglia to achieve relief from memory loss in the Alzheimer’s Disease mouse model (Xiao et al., 2024). Furthermore, creatinine protects the nervous system from oxidative stress by upregulating significant anti-oxidant enzymes. Creatinine as an anti-inflammation factor may reduce endothelial permeability and the expression of Toll-like Receptor 2 (TLR2) (Chang and Leem, 2023). However, several studies discredit the value of creatinine as a biomarker in the diagnosis and progression of neurodegenerative diseases such as amyotrophic lateral sclerosis (Lv and Li, 2025) and Alzheimer’s disease (Binette et al., 2022).

Recent studies identified different categories of predictors for PSCI. Hs-CRP is one of the most extensively studied factors that changes may be associated with neurodegenerative disease, including cognitive decline after stroke (Wang et al., 2023; Baba and Yarube, 2021; Zhang and Bi, 2020). Similar to our research, Hs-CRP is correlated but not yet proven an independent risk factor for early-onset PSCI. Compared with similar metrics, like UAR (the uric acid to albumin ratio) and NLR (the neutrophil-to-lymphocyte ratio), SUA/SCr has a more striking predictive value in predicting neurological disorders, especially in patients with acute ischemic stroke (Liu et al., 2025). UAR as an inflammation marker, shows its wonderful value in predicting cardiovascular disease, but insufficient evidence supports that UAR correlates with cognitive status (Klisic et al., 2025; Zhang et al., 2024). The NLR has been widely studied and showed that the NLR is strongly associated with various types of tumors (Mouchli et al., 2021; Saputra et al., 2022). In contrast, the relationship between SUA/SCr and cognitive function has been confirmed (Chen et al., 2024; Zhou et al., 2025). Therefore, SUA/SCr may be a metric worth exploring in further detail. Combining SUA/SCr with other indicators or factors may improve the prediction to PSCI.

Numerous risk factors contribute to PSCI, and educational level is one of them. Individuals who have attained a high level of education are better compensated for brain injury, thereby maintaining good cognitive function. There is an inverse association between education level and the degree of risk of cognitive impairment after stroke (Shin et al., 2024). We also found the education level differs between groups PSCI and Non-PSCI in this study. Interestingly, lymphocytes were significantly different in PSCI and non-PSCI groups, different SUA/SCr tertiles, and associated with early-onset PSCI. Considering that lymphocytes may be the confounders, we adjusted them when developing models to instigate the relationship between SUA/SCr and early-onset PSCI. Emerging study proves that lymphocytes exactly correlate with cognitive abilities and stroke. T lymphocytes can infiltrate the brain parenchyma contributing to age-associated cognitive impairment. Circulating T cells increased and circulating B cells decreased after stroke, and these changes were associated with poor stroke outcomes (Chauhan and Nguyen, 2023). In addition, T cells play a vital role in mediating neurodegenerative and cognitive decline by upsetting cellular homeostasis, inducing synaptic plasticity damage, encouraging Aβ deposition, and releasing pro-inflammatory factors (Zeng et al., 2024). Further study is warranted on the relationship between lymphocytes and early-onset PSCI.

Additionally, some key clinical variables might have influenced our outcomes. The relationship between brain lesion location and cognition is strongly correlated, although our study did not analyze it. A large-scale multicenter study showed that infarcts in the left frontotemporal regions, the right parietal lobe, and the left thalamus were strongly associated with PSCI (Weaver et al., 2021). It should be noted that the volume of the infarct and the location of the stroke are also significantly associated with PSCI (Pendlebury and Rothwell, 2009). But the cognitive declines did not differ by stroke type (ischemic stroke and hemorrhagic stroke) or ischemic stroke etiology (e.g., large artery atherosclerotic/cardioembolic) (Levine et al., 2025). The electrolyte status may affect the serum uric acid to serum creatinine ratio. High levels of serum potassium may be associated with elevated SUA levels (Zhang et al., 2025), abnormal serum sodium, such as dysnatremia, hyponatremia, and hypernatremia are significantly associated with acute kidney injury, therefore leading to an increase in creatinine levels (Lombardi et al., 2020). Antihypertensive or urate-lowering therapy can also affect study outcomes. Reduced serum uric acid levels after uric acid-lowering therapy will certainly cause changes in the serum uric acid to serum creatinine ratio. A cross-sectional study of 1,000 participants revealed that subjects with elevated SUA levels had a higher proportion of antihypertensive medication users, and antihypertensive medications may be associated with SUA levels (Tang et al., 2022).

## 5 Limitation

To our knowledge, our study filled the knowledge gap by demonstrating the probable link between serum uric acid to serum creatinine ratio and cognitive decline in acute ischemic stroke. There were several limitations in our study. Firstly, this was a single-center observational study, therefore, the generalization of the results was limited to some extent. Further studies are needed to validate these biomarkers through large-sample multi-center trials. Secondly, our study population comprised a small number of patients for various reasons, which limited our ability to explore the relationship between SUA/SCr and PSCI in greater depth. It is recommended that the sample size be further expanded and a multi-center collaborative study be conducted in future research work. Third, the serum uric acid and creatinine levels were measured once, ignoring possible intra-individual fluctuations. Fourth, our study focused only on the evaluation of cognitive function after acute stroke and did not perform baseline testing and long-term follow-up of patients. While the cognitive status changes considerably at acute and chronic stroke periods, further larger and more diverse cohorts with stronger validity are urgently needed to validate our results. Fifth, we excluded the patients with dementia before the stroke, but we did not test their cognitive status. Those individuals with severe cognitive dysfunction or who had aphasia could not complete the cognitive assessment. Thus, the relationship between SUA/SCr levels and severe cognitive decline is unclear, leading to limited generalization. Sixth, our study does not provide sufficient statistical power due to the small sample size to some extent. Multivariate adjustments are needed to exclude the many factors that confound the results, whereas each of the three stratified models compares only a few dozen patients per group, which raises concerns about overfitting inevitably. In addition, despite our efforts to adjust for numerous factors that could influence the results, there are still many confounders, such as lesion location and volume, comorbidities, and medication history remained uncontrolled. The above factors have led to limitations in statistical methods; large-sample and follow-up studies may be able to optimize them. Seventh, the AUC of SUA/SCr obtained by ROC analysis in this study was 0.677 for predicting PSCI, which is only of medium predictive power, and the predictive value is not outstanding. Its actual clinical feasibility needs to be further explored. Meanwhile, SUA/SCr was not modeled jointly with other indicators, and subsequent studies could combine other factors for comprehensive prediction to improve the prediction effect. Lastly, the dynamic changes in SUA/SCr were not recorded. The cognitive ability after stroke may have been affected by changes in SUA/SCr during the follow-up period. Combining the admission SUA/SCr with dynamic changes would be better, which would provide better prognostic information.

## 6 Conclusion

In conclusion, our study found SUA/SCr ratio upon admission was an independent predictor of cognitive impairment in the early stage after stroke and a protective factor against early post-stroke cognitive impairment, meaning that individuals with lower SUA/SCr values have an increased risk of developing post-stroke cognitive impairment. The results expand the field of assessing PSCI, especially in the acute phase of stroke. Further prospective studies are needed to verify the potential mechanisms underlying this relationship.

## Data Availability

The raw data supporting the conclusions of this article will be made available by the authors without undue reservation.
